# The Effect of Yiqi Huoxue Tongluo Decoction on Spinal Cord Microglia Activation and ASK1-MKK3-p38 Signal Pathway in Rats with Diabetic Neuropathic Pain

**DOI:** 10.1155/2022/2408265

**Published:** 2022-05-19

**Authors:** Fanjing Wang, Heyong Tang, Junlong Ma, Lianzhi Cheng, Yixuan Lin, Jindong Zhao, Guoming Shen, Zhaohui Fang, Aijuan Jiang

**Affiliations:** ^1^Department of Endocrine, First Affiliated Hospital of Anhui University of Chinese Medicine, Hefei 230031, China; ^2^School of Integrated Chinese and Western Medicine, Anhui University of Chinese Medicine, Hefei 230012, China; ^3^Anhui Jing'an Hospital of Integrated Traditional Chinese and Western Medicine, Hefei 230011, China; ^4^Diabetes Institute, Anhui Academy of Chinese Medicine, Hefei 230038, China; ^5^Key Laboratory of Xin'an Medicine, Ministry of Education, Hefei 230038, China

## Abstract

Diabetic neuropathic pain (DNP) is one of the most common chronic peripheral neuropathies in diabetes mellitus (DM). *Objective*. To observe the underlying mechanism of the effects of Yiqi Huoxue Tongluo Decoction (YQHX) on DNP rats. *Methods*. SD rats were intraperitoneally injected with 35 mg/kg streptozotocin (STZ) to prepare DNP models and were treated with YQHX for 8 weeks. *Results*. Studies have shown that the drug restores some levels of MWT, TWL, and MNCV, downregulates the levels of inflammatory factors IL-6, IL-1*β*, and TNF-*α*, downregulates the levels of ASK1-MKK3-p38, and weakens the level of OX42 activation. *Conclusion*. Yiqi Huoxue Tongluo Decoction can relieve DNP by affecting the activity of spinal cord microglia and the ASK1-MKK3-p38 signaling pathway, thereby reducing the central sensitization caused by the inflammatory response of DNP rats.

## 1. Introduction

DNP is one of the most common chronic complications of diabetes mellitus (DM). Clinically, about 50% of diabetic patients are accompanied by diabetic neuropathy and 20%–30% of them are concomitant with DNP [1–3]. The clinical manifestations of DNP are spontaneous pain, hyperalgesia, and allodynia. Neuropathic pain is caused by primary damage or dysfunction of the nervous system [4]. In the theory of Chinese medicine, DM belongs to the category of “diarrhea,” while DNP has symptoms such as numbness and pain in the extremities due to the long-term dysthymia, blood stasis, and obstruction of the collaterals. The pathological process of Qi deficiency-yin deficiency-blood stasis is consistent with diabetic peripheral nerve inflammation and hyperalgesia. At present, DNP is a common disabling complication of DM [5] and its clinical efficacy is not optimistic. Therefore, elucidating the mechanism of pain hypersensitivity caused by nerve injury is essential for the treatment of DNP.

The pathogenesis of DNP is caused by the sensitization of the peripheral nervous system and central nervous system (CNS) [6]. Spinal cord microglia, as inherent phagocytes in the central nervous system, account for about 5%–20% of the total cell population [7]. They respond to stimuli that threaten the body's physiological homeostasis and activate them into an activated state. The immune environment plays an important role and is closely related to chronic neuroinflammatory diseases [8]. The activation of spinal cord microglia can be judged by the expression of OX42, so OX42 can be used as a specific marker to identify spinal cord microglia.

p38-MAPK is one of the three classic subsystems of the mitogen-activated protein kinases (MAPKs) signaling pathway [9], which can mediate various cellular functions such as immunity, inflammation, proliferation, differentiation, and migration [9, 10]. The ASK1-MKK3-p38 signaling pathway is a cascade pathway. ASK1 can activate the p38-MAPK pathway by activating MKK6 and MKK3. Studies have shown that p38-MAPK is closely related to the occurrence and development of neuropathic pain. As an intracellular signal transduction pathway and transduction molecule, it plays an important role in the release of inflammatory factors [11]. The activation of p38-MAPK plays a vital role in the occurrence of DNP [12]. Spinal cord and dorsal root ganglion neurons in DNP rats induced by streptozotocin (STZ) may develop neurological deficits. The obstacle is related to the increase of phosphorylated p38-MAPK [13, 14], which can affect the process and outcome of DNP by improving the insulin signal transduction system [15], mediating cell senescence and apoptosis [16], participating in immune responses, etc. In the experiment, inhibitors can be used to inhibit the phosphorylation of MAPK by reducing the activation of microglia and further reduce mechanical hyperalgesia in diabetic rats [17]. Therefore, regulating the p38-MAPK signal transduction pathway, blocking the activation of spinal cord microglia, and reducing the inflammatory response may be potential targets for the prevention and treatment of DNP.

This topic uses the in-hospital preparation “Yiqi Huoxue Tongluo Decoction” from the First Affiliated Hospital of Anhui University of Traditional Chinese Medicine. Under the guidance of Xin'an medical theory, the decoction is made according to the therapeutic principles of invigorating qi and nourishing yin, promoting blood circulation, and removing blood stasis. Its ingredients include 30 g of *Astragalus*, 12 g of *Angelica*, 12 g of *Rehmannia glutinosa*, 9 g of *Corydalis*, 12 g of *Pueraria lobata*, 15 g of *Caulis spatholobi*, and 9 g of Weilingxian. The effect of this decoction on spinal cord microglia in DNP has not been reported. Therefore, this project will deeply explore the role of Yiqi Huoxue Tongluo Decoction in the inflammatory response of the central nervous system and its relationship with spinal cord microglia activity, and the interactive regulation relationship with the ASK1-MKK3-p38 signaling pathway provides a theoretical basis for reasonable clinical application.

## 2. Experimental Methods and Animals

58 SPF male SD rats, weighing 180–220 g, were purchased from Nanjing Qinglongshan Animal Farm. They were fed adaptively for one week and kept in an environment with a humidity of 36%, a temperature of 25°C, and alternating day and night for 12 hours. Food and water were continuously available. Animal production license number: SCXK (Zhejiang) 2018-0001.

### 2.1. Establishment and Administration of DNP Model

After 7 days of adaptive feeding, 10 rats in the normal group (Control) were selected according to the principle of random control. They were fed with a regular diet, and the remaining 48 rats were fed with a high-fat diet. At the end of the 4th week, rats in the control group were injected intraperitoneally with citric acid solution.

In addition, the remaining rats were intraperitoneally injected with streptozotocin (STZ) at 35 mg/kg. Tail vein blood was collected after 72 h. A type 2 diabetes model was established when the fasting blood glucose (FBG) ≥11.1 mmol/L. The basic value of mechanical withdrawal threshold (MWT) and the thermal withdrawal latency (TWL) was detected. Two weeks later, MWT and TWL were tested, and the rats that both fell below 85% of the baseline value can be judged as DNP model rats. Excluding nondiabetic models and dead rats, a total of 30 rats finally created DNP models, with a comprehensive model rate of 62.5%.

### 2.2. Animal Group Administration and Drug Configuration

After the DNP model was modeled, the rats were gavaged and the model rats were divided into 3 groups according to the principle of random control, namely, the model group (Model), the group of Yiqi Huoxue Tongluo Decoction (YQHX), and Positive Control of methylcobalamin. Before administration and at the 8th week of administration, blood was collected from the tail vein to measure FBG, the YQHX group was given gavage at 4.5 g/kg, and for the Positive Control group, methylcobalamin (0.175 mg/kg) was given by gavage, and the Model group and Control group were given the same amount of distilled water by gavage for 8 weeks.

YQHX is made up of 20 g of *Astragalus*, 12 g of *Angelica*, 15 g of Radix Corydalis, 12 g of *Corydalis*, 10 g of Weilingxian, 10 g of *Caulis spatholobi*, and 10 g of *Pueraria lobata* root. It is an in-hospital preparation of the First Affiliated Hospital of Anhui University of Traditional Chinese Medicine. The YQHX group was administered 9 times the clinically equivalent dose. The Positive Control group dissolved mecobalamin tablets in pure water to make a suspension for intragastric administration.

### 2.3. Mechanical Withdrawal Threshold (MWT) Measurement

The von Frey fiber pain meter was used to detect MWT at week 0 (starting gastric administration) and 2, 4, 6, and 8 weeks. The rat was placed on a sieve-shaped metal frame and fixed in a transparent glass cover. After about 30 minutes, the rat was quiet. The von Frey fiber needle was used to stimulate the rat's right hind foot vertically. It was regarded as a positive reaction when the rat raised legs, licked feet, or avoided them. 5 times tests were carried out in a row with an interval of 15 s between each measurement. The minimum number of grams of each positive reaction was recorded. The average of 5 times was taken as the MWT of the rat.

### 2.4. Thermal Withdrawal Latency (TWL) Measurement

TWL was tested with a hot plate instrument at week 0 (starting gastric administration) and 2, 4, 6, and 8 weeks. The RB-200 intelligent thermal instrument was warmed up to 55°C and controlled at the constant temperature. The moment when the rat was placed in the thermal instrument and the moment the rat's hindlimbs retracted or the rat licked the hindlimbs were recorded. The time difference between the two moments was used as an indicator of pain response, that is, the pain threshold. Each rat was given 3 heat stimulations with 15 minutes as the interval to take 3 times of response time. The average value is the TWL of the rat.

### 2.5. Motor Nerve Conduction Velocity (MNCV) Measurement

After anesthesia, the rats were fixed in the prone position to expose the sciatic nerve. The needle is then inserted into the proximal sciatic nerve. After the stimulation electrodes were attached, the needle was inserted into the distal end. The receiving electrode was connected to the tibial peroneal nerve. Then, PowerLab 8/30 biological information acquisition system was started to collect the motor nerve conduction velocity (MNCV). MNCV = distance between two electrodes (m)/difference in action potential latency (s).

### 2.6. Specimen Collection and Processing

At the end of the experiment, the rats were anesthetized by intraperitoneal injection of sodium pentobarbital. After general anesthesia, the rats were fixed in a supine position. Abdominal cavity of the rat was opened with surgical scissors. After blood was collected from the abdominal aorta, the L4-6 spinal cord was surgically cut from the lumbosacral enlargement and frozen in a −80°C refrigerator for WB and ELISA measurements. After the rats were anesthetized and their blood was collected, they were frozen and fixed. Their hearts were perfused with normal saline until the blood of the effluent was clear, followed by 4% paraformaldehyde perfusion. After fixation, the tissues were placed in 4% paraformaldehyde for 12 hours.

### 2.7. ELISA

According to the manufacturer's instructions, the concentrations of IL-6, IL-1*β*, and TNF-*α* in the homogenate of rat spinal cord L4-6 were detected by using enzyme-linked immunosorbent assay kit (Yiyan, China).

### 2.8. qRT-PCR

Total RNA samples from the spinal cord were isolated with TRIzol reagent (Invitrogen, USA). RNA samples from total RNA were reverse transcribed to cDNA. qRT-PCR was analyzed by using the CFX-Connect96 Real-Time PCR detection instrument (Bio-Rad, USA). Primers for ASK1, MKK3, p38, OX42, and *β*-actin used in this experiment are sequenced in Table 1. *β*-Actin was used as an internal control. The relative mRNA expression levels were quantified using the 2^−ΔΔCq^ method.

### 2.9. Western Blot Analysis

Protein extracts of rat spinal cord tissue L4-6 were added to a homogenizer. The supernatant was obtained by centrifugation after thorough trituration. SDS protein loading buffer was added to the supernatant to extract protein samples. Target proteins of different molecular weights were obtained by electrophoresis. After being attached to the NC protein membrane, the membrane was transferred and blocked. Membranes were washed 3 times with TBS before being transferred to primary antibodies and incubated overnight at 4°C. The p38 antibody (Abcam, USA), p-p38 antibody (Abcam, USA), OX42 antibody (Abcam, USA), ASK1 (Abbkine, China), MKK3 (Abbkine, China), and *β*-actin antibody (Abcam, USA) were added, respectively. After washing, the membranes were incubated in secondary antibody and rinsed with TBS solution 3 times. The ECL working droplets were added to the surface of the NC membrane, and then the membrane was placed in an FCM gel imager for exposure and photography.

### 2.10. Immunofluorescence

The rat spinal cord L4-6 was dehydrated through a sucrose gradient. The tissues were surrounded by OCT, and then dry ice and absolute ethanol were poured into the Petri dish to quick-freeze the tissue. After quick freezing, place the tissue on the microtome and cut out 15 *μ*m tissue sections. The experimental area was circled with an immunohistochemical pen, fixed with PFA for 30 min, and blocked with goat serum for 30 min. Shake off the goat serum, drop the primary antibody p-p38 (Abcam, USA) and OX42 (Abcam, USA), and incubate at 4°C for one night. The next day, rinse with PBST. Add fluorescent secondary antibody, incubate at 37°C for 30 min in the dark, and then rinse with PBST. After counterstained by DAPI, wash with PBST 3 times. The slides were mounted with antifluorescence quenching mounting medium and photographed by using a fluorescence microscope.

### 2.11. Statistical Methods

SPSS 25.0 software was used for statistical analysis, and the data were expressed as $\bar{x}$ ± *s*. One-way ANOVA was used for comparison of multiple groups. The LSD method was used for homogeneity of variance, and Dunnett's test was used for heterogeneity of variance. MWT and TWL were analyzed using a two-way analysis of variance (ANOVA) with repeated measures followed by Turkey's post-hoc honestly significant difference (HSD) test. *P* < 0.05 was considered statistically significant. WB and immunofluorescence were analyzed using Image J. The results were analyzed by using GraphPad Prism 8 graphically.

## 3. Results

### 3.1. Effects of Yiqi Huoxue Tongluo Decoction on FBG in Rats

**Figure 1:**
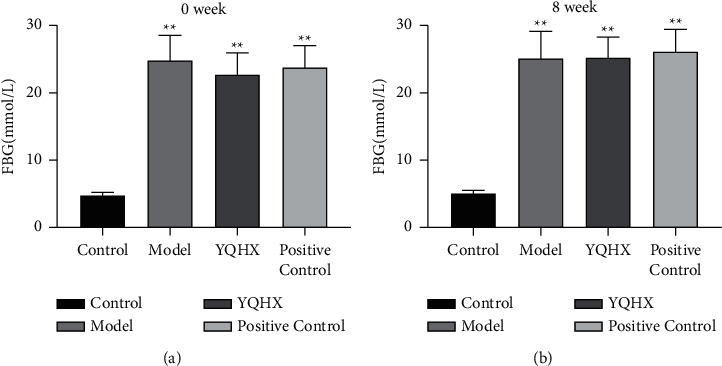
Effects of Yiqi Huoxue Tongluo Decoction on FBG in rats. (a) Effect of YQHX on blood glucose in rats before administration. (b) Effects of YQHX on blood glucose in rats after 8 weeks of administration. ^*∗∗*^*P* < 0.01 vs. Control.

Before administration, compared with the Control group, the FBG of the Model group increased (*P* < 0.01). While compared with the Model group, there was no significant difference in the YQHX and Positive Control groups (*P* > 0.05) (Figure 1(a)). In the 8th week, compared with the Control group, the FBG of the Model group increased (*P* < 0.01). While compared with the Model group, in the YQHX or Positive Control group, the difference was not statistically significant (*P* > 0.05) (Figure 1(b)).

### 3.2. Yiqi Huoxue Tongluo Decoction Relieves DNP-Induced Pain in DNP Rats

**Figure 2:**
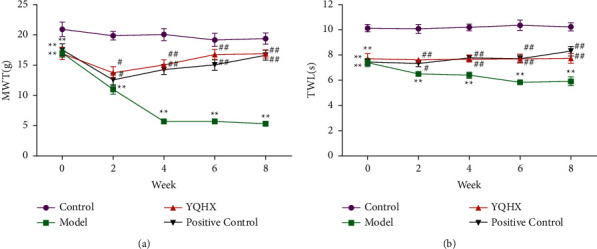
Yiqi Huoxue Tongluo Decoction relieves DNP-induced pain in rats. (a) The effect of YQHX on DNP-induced mechanical pain threshold. (b) The effect of YQHX on DNP-induced thermal withdrawal latency. ^∗∗^*P* < 0.01 vs. Control; ^#^*P* < 0.05 and ^##^*P* < 0.01 vs. Model.

We studied the effects of YQHX on MWT and TWL in DNP rats at different time periods. The mechanical withdrawal threshold (MWT) measurement and thermal withdrawal latency (TWL) measurement were examined at 4 groups at 5 time points (time-course) and evaluated by a two-way ANOVA with repeated measures (treatment × time). MWT: *F*(3,27) = 102.906, *P* < 0.001, and *F*(4,36) = 19.949, *P* < 0.0001, for the treatment and time factors, respectively; interaction factor: *F*(12,108) = 10.023, *P* < 0.001. TWL: *F*(3,27) = 82.677, *P* < 0.001, and *F*(4,36) = 1.021, *n.s.*, for the treatment and time factors, respectively; interaction factor: *F*(12,108) = 1.738, *n.s*. Tukey's post-hoc HSD tests showed that the MWT and TWL decreased at 2, 4, 6, and 8 weeks after YQHX administration, in comparison to the Model group. Before administration, compared with the Control group, the MWT and TWL values of the other groups were significantly decreased (*P* < 0.01). In the second week, compared with the Control group, the MWT and TWL of the Model group were decreased (*P* < 0.05). Compared with the Model group, the MWT and TWL of the YQHX group were significantly different (*P* < 0.05 and*P* < 0.01). At the 4th, 6th, and 8th weeks, compared with the Model group, the MWT and TWL of the YQHX group and the Positive Control group were significantly different (*P* < 0.01). It shows that Yiqi Huoxue Tongluo Decoction has an obvious analgesic effect on DNP rats (Figure 2).

### 3.3. Yiqi Huoxue Tongluo Decoction Improves MNCV in DNP Rats

**Figure 3:**
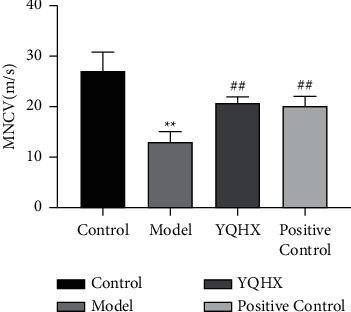
Yiqi Huoxue Tongluo Decoction improved MNCV in DNP rats. ^∗∗^*P* < 0.01 vs. Control; ^#^*P* < 0.05 and ^##^*P* < 0.01 vs. Model.

We further investigated the effect of YQHX on MNCV in DNP rats. Compared with the Control group, the MNCV of the Model group decreased, and the difference was statistically significant (*P* < 0.01). Compared with the Model group, the MNCV of the YQHX and Positive Control groups increased, and the difference was statistically significant (*P* < 0.01) (Figure 3).

### 3.4. Yiqi Huoxue Tongluo Decoction Alleviates Inflammation in DNP Rats

**Figure 4:**
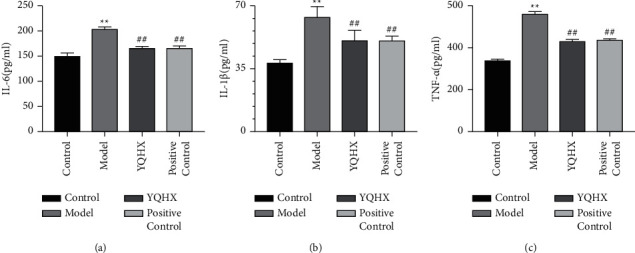
Yiqi Huoxue Tongluo Decoction alleviates inflammation in DNP rats. (a) IL-6, (b) IL-1*β*, and (c) TNF-*α* by ELISA. ^∗∗^*P* < 0.01 vs. Control; ^#^*P* < 0.05 and ^##^*P* < 0.01 vs. Model.

Compared with the rats in the Control group, the levels of IL-6, IL-1*β*, and TNF-*α* in the spinal cord homogenate of the rats in the Model group were increased, and the difference was statistically significant (*P* < 0.01). The levels of IL-6, IL-1*β*, and TNF-*α* in the spinal cord homogenate in the YQHX and Positive Control groups were decreased, and the differences were statistically significant (*P* < 0.01) (Figure 4).

### 3.5. Effect of Yiqi Huoxue Tongluo Decoction on Relative mRNA Level of ASK1, MKK3, p38, and OX42 in DNP Rats

**Figure 5:**
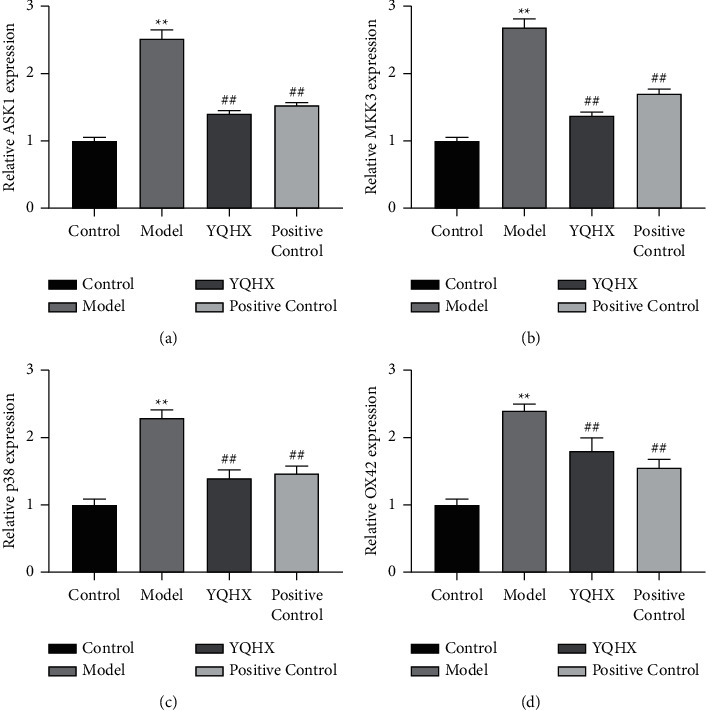
Effect of Yiqi Huoxue Tongluo Decoction on relative mRNA expression of ASK1 (a), MKK3 (b), p38 (c), and OX42 (d) in DNP rats. ^∗∗^*P* < 0.01 vs. Control; ^#^*P* < 0.05 and ^##^*P* < 0.01 vs. Model.

Compared with the Control group, the mRNA expressions of ASK1, MKK3, p38, and OX42 in the Model group were increased (*P* < 0.01). Compared with the Model group, the mRNA expressions of ASK1, MKK3, p38, and OX42 in YQHX and Positive Control groups were decreased (*P* < 0.01). The results showed that YQHX could significantly downregulate the relative mRNA expression of ASK1, MKK3, p38, and OX42 in DNP rats (Figure 5).

### 3.6. Yiqi Huoxue Tongluo Decoction Reduces the Protein Expression on OX42, ASK1, MKK3, p38, and p-p38 in the Spinal Cord of DNP Rats

**Figure 6:**
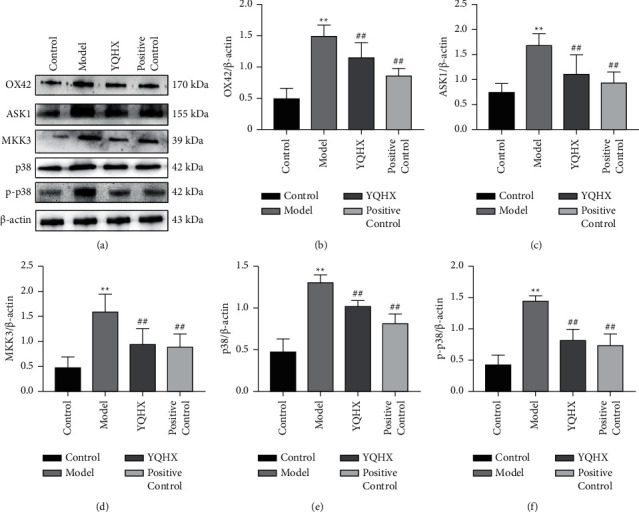
Yiqi Huoxue Tongluo Decoction reduces the expressions of OX42 (b), ASK1 (c), MKK3 (d), p38 (e), and p-p38 (f) in the spinal cord of DNP rats. ^*∗∗*^*P* < 0.01 vs. Control; ^#^*P* < 0.05 and ^##^*P* < 0.01 vs. Model.

The results of WB showed that compared with the rats in the Control group, the protein expressions of OX42, ASK1, MKK3, p38, and p-p38 in the Model group were increased (*P* < 0.01). Compared with the Model group, the protein expressions of OX42, ASK1, MKK3, p38, and p-p38 in YQHX and Positive Control groups were decreased, and the difference was statistically significant (*P* < 0.01) (Figure 6). The results showed that YQHX could significantly downregulate the expressions of OX42, ASK1, MKK3, and p38 in DNP rats.

### 3.7. Yiqi Huoxue Tongluo Decoction Reduces the Fluorescence Expression of p-p38 and OX42 in DNP Rats

**Figure 7:**
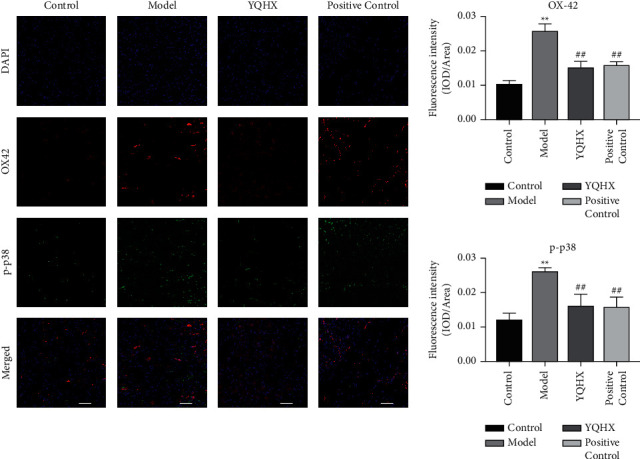
Effects of Yiqi Huoxue Tongluo Decoction on p-p38-MAPK and OX42 expression in DNP rats.^∗∗^*P* < 0.01 vs. Control; ^#^*P* < 0.05 and ^##^*P* < 0.01 vs. Model.

Compared with the rats in the Control group, the expressions of p-p38 and OX42 in the Model group were increased (*P* < 0.01), and the number of microglia cells was increased. Compared with the Model group, the expressions of p-p38 and OX42 in the YQHX and Positive Control groups were decreased (*P* < 0.01) and the number of microglia cells was decreased. The results showed that YQHX could significantly downregulate the fluorescence expression of p-p38 and OX42 in DNP rats (Figure 7).

## 4. Discussion

In this study, by observing the effect of Yiqi Huoxue Tongluo Decoction (YQHX) on the activity of spinal cord microglia mediated by the ASK1-MKK3-p38 signaling pathway in DNP rats and on neural hyperalgesia, the mechanism of neuroprotective effect of YQHX on DNP was discussed. It will provide further experimental basis for the clinical application and promotion of YQHX.

**Figure 8:**
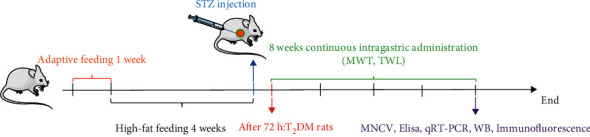
The research route of this experimental process.

DNP is a chronic low-grade neuroinflammatory response [18, 19]. An effective DNP model can be established by STZ. STZ can selectively destroy pancreatic islet *β* cells to induce DM, which is widely used in the field of endocrinology. Here, a high-fat diet combined with intraperitoneal injection of low-dose STZ (35 mg/kg) was used to establish a DM rat model. DNP model rats were screened out according to pain threshold. At first, 10 rats out of 58 rats were selected to establish the Control group. The DNP model was established by 30 rats out of the rest 48 rats, except those that did not form a model or died. The comprehensive model rate was 62.5%. It was confirmed that this method established a relatively stable DNP rat model [20]. The research route of this experimental process was depicted by a flowchart (Figure 8).

Traditional Chinese Medicine can effectively improve symptoms through multiple channels and multiple targets [21–24]. The treatment of DNP in Traditional Chinese Medicine is based on syndrome differentiation and overall treatment. Modern pharmacological studies have found that *Astragalus* can reduce blood sugar, improve blood rheology, improve microcirculation, and promote the growth and repair of nerve cells [25]. *Angelica sinensis* and Shengdi decoction have significant inhibitory effects on acute and chronic inflammation caused by various inflammatory agents [26]. *Sophora japonica* has the effect of improving hemodynamics, regulating immunity, and increasing white blood cells [27]. A variety of saponins purified from Weilingxian have analgesic and sedative effects [28]. Studies have shown that Traditional Chinese Medicine compounds can improve diabetic peripheral nerve damage by inhibiting the inflammatory response factors of diabetes [29]. Methylcobalamin has been shown to be beneficial in relieving neuropathic pain symptoms and improving nerve conduction [30, 31] and is often used to alleviate diabetic neuropathy, improve nerve conduction, and promote regeneration of damaged nerves. Based on these studies, we speculate that Yiqi Huoxue Tongluo Decoction might be an effective drug for DNP. However, the potential mechanism of YQHX remains to be further studied.

If the body is in a pathological state of high glucose for a long time, it will lead to metabolic disorders. Biochemical indicators such as blood lipids will be abnormal. Blood viscosity will be increased. The capillaries will be blocked, along with the destruction of functional structures, leaving the nerves deprived of nourishment. This in turn induces neuromotor and sensory disturbances, resulting in lower thresholds and lower nerve conduction velocity. Studies have shown that the Traditional Chinese Medicine *Astragalus* can improve the microcirculation in rats, promote the regeneration of nerve cells, and has a protective effect on the central nervous system [32]. In our study, YQHX was found to be able to improve MWT and TWL and prolong MNCV compared with the Model group, indicating that YQHX can improve DNP without affecting blood glucose levels.

Inflammatory factors play an important role in painful neuropathy [33]. Inflammatory factors such as IL-6, IL-1*β*, and TNF-*α* can damage glycosylated myelin proteins, demyelinate nerves, and cause central sensitization, which in turn leads to pain. In this study, the levels of IL-6, IL-1*β*, and TNF-*α* increased in DNP rats. Unlike cytokine antagonists, which block only one specific inflammatory cytokine, Chinese herbal medicines tend to affect the production of multiple cytokines, reduce excessive inflammatory responses, and produce relatively few side effects [34]. In this experiment, ELISA results showed that the concentrations of proinflammatory cytokines IL-6, IL-1*β*, and TNF-*α* in spinal cord tissue decreased after YQHX treatment. We speculate that YQHX may improve DNP, possibly partly due to its anti-inflammatory effect. The mechanism of the protective effect of Yiqi Huoxue Tongluo Decoction on DNP central sensitization was preliminarily discussed.

Studies have shown that spinal cord microglia can be activated in a variety of neuropathic pain models, transforming from “resting state” to “activated state” [35] that can actively function. It is manifested by morphological changes and the upregulation of OX42 expression [36]. The spinal cord is the primary integration center of sensation. When neuroinflammatory injury occurs, microglia in the peripheral and central systems can be activated to varying degrees. Noxious information is continuously transmitted to the center from the periphery.

Microglia respond to extracellular stimuli through transduction of intracellular signaling cascades such as ASK1-MKK3-p38 [13], which can be activated to varying degrees to produce various immune cells and inflammatory mediators. The mediated proinflammatory cytokines and chemokines have become important pain-inducing factors in the occurrence and development of neuropathic pain. Spinal cord microglia activation is the major source of inflammatory factors IL-6, IL-1*β*, and TNF-*α*, which can strongly respond to neuronal injury and degeneration [37]. In our study, OX42 was overexpressed in the Model group. Compared with the Model group, the protein and mRNA levels of OX42 in DNP rats were decreased in both the YQHX group and the Positive Control group (*P* < 0.01). However, after YQHX treatment, the expression of OX42 was significantly inhibited. While spinal cord microglia were inhibited and the expression of inflammatory factors was reduced, there was no significant change in blood glucose in rats. These results suggest that YQHX can alleviate DNP. The mechanism may be related to the inhibition of spinal cord microglia and the regulation of inflammatory factors.

Studies have shown that p38-MAPK plays an important role in regulating inflammation, cell differentiation, cell growth, and cell death [38]. During the development of DNP, activated p38-MAPK regulates a variety of transcription factors through its phosphorylation. The p38-MAPK upstream kinase activator is induced to increase. Genes for proinflammatory molecules such as IL-1*β*, IL-6, and TNF-*α* are encoded and expressed [39–41]. The upstream-specific kinase MKK3 of p38-MAPK belongs to the MAPKK family and can activate p38-MAPK to upregulate its expression [42]. ASK1 can activate MAPKK, leading to neuronal death. Thus, the activation of inflammatory cascade plays a key role in the development and persistence of neuropathic pain [43].

**Figure 9:**
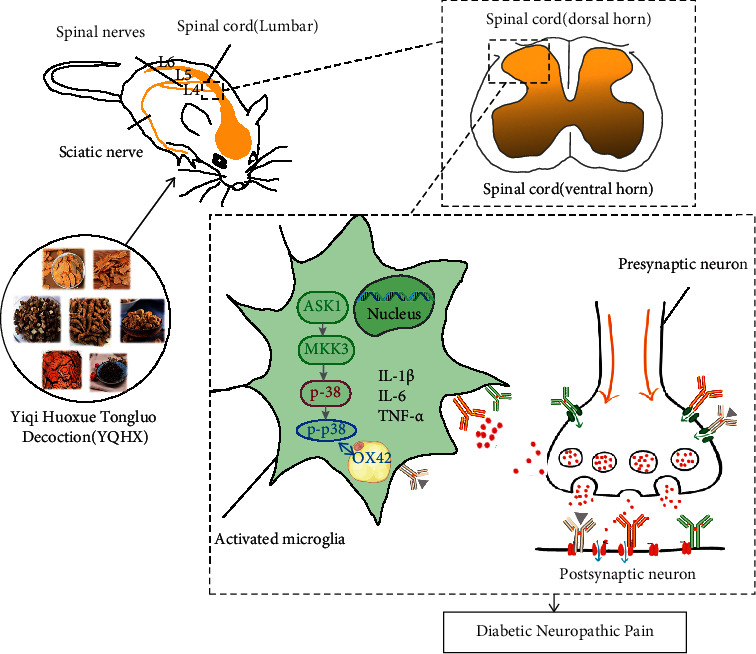
Schematic diagram depicting the effects of YQHX on diabetic neuropathic pain via inhibiting the ASK1-MKK3-p38 signaling pathway in spinal cord microglia.

Under the stimulation of nerve injury, the activated spinal cord microglia can initiate the secretion of inflammatory factors through the p38-MAPK signaling pathway. The inflammatory response can also increase the activity of p38-MAPK. The activation of spinal cord microglia is promoted, resulting in the formation of a positive feedback pathway between inflammatory factors and microglia. ASK1, an apoptotic signal-regulated kinase, is one of the MAPKKKs and is an upstream factor that activates the MKK3-p38 signaling cascade [44]. In this study, we found that, after the treatment of YQHX, compared with the Model group, YQHX can reduce the protein and mRNA expressions of ASK1, MKK3, and p38 in DNP rats. OX42 is the antigen substance of spinal cord microglia; the expression of OX42 can be used as a specific marker to identify whether microglia are activated [45, 46]. In this work, we studied the effect of Yiqi Huoxue Tongluo Decoction on spinal cord microglia and the corresponding correlation with ASK1-MKK3-p38 signaling pathway. By observing the expression of p38 signaling pathway and its upstream MKK3, ASK1 signaling pathway-related proteins, mRNA, and fluorescence, it can be inferred that the alleviation of DNP by YQHX is related to the regulation of the inhibition of ASK1-MKK3-p38 signaling pathway in spinal cord microglia [47, 48]. The effects of YQHX on diabetic neuropathic pain via inhibiting the ASK1-MKK3-p38 signaling pathway in spinal cord microglia were depicted by schematic diagram (Figure 9).

## 5. Conclusion

The results of this study show that high-fat diet combined with intraperitoneal injection of STZ can induce DNP rat model and Yiqi Huoxue Tongluo Decoction can improve pain threshold and MNCV in DNP rats. Yiqi Huoxue Tongluo Decoction may relieve DNP by inhibiting the abnormal activation of ASK1-MKK3-p38 signaling pathway, attenuating the proliferation and activation of spinal cord microglia. The release of proinflammatory factors is inhibited, thereby reducing the central sensitization caused by the inflammatory response in DNP rats. Overall, these findings are providing new ideas for the development of therapeutic DNP drugs based on eliminating the potentially central sensitization.

## Figures and Tables

**Table 1:** Primers used in quantitative real-time PCR.

Gene	Forward primer (5′–3′)	Reverse primer (5′–3′)
ASK1	CAGGATGCGGTCAATAAAGT	GCGAGGCTGAAATGTGG
MKK3	CTGCGATTCCCTTACGAGT	GTCCGTCTTCTTAGTTTTGTGC
p38	AGCAACCTCGCTGTGAATG	ACAACGTTCTTCCGGTCAAC
OX42	CAAGGAGTGTGTTTGCGTGTC	TGAGTATGCCGTTCTTTGTTTC
*β*-Actin	CGTTGACATCCGTAAAGAC	TAGGAGCCAGGGCAGTA

## Data Availability

The figures and tables supporting the results of this study are included in the article, and the original datasets are available from the first author or corresponding author upon request.
